# Data sparse inference of operator spatial reward models in uncertain environments

**DOI:** 10.3389/frobt.2026.1751002

**Published:** 2026-03-26

**Authors:** Hunter M. Ray, Aditya Pandey, Nisar Ahmed

**Affiliations:** Cooperative Human-Robot Intelligence Laboratory, Department of Aerospace Engineering, University of Colorado Boulder, Boulder, CO, United States

**Keywords:** autonomous aerial vehicles, autonomy systems, graphical models, human–machine systems, human–robot interaction, rescue robots

## Abstract

Human–machine teaming allows people to leverage the impressive capabilities of autonomous robotic teammates to safely accomplish challenging tasks. Although users may be experts in their fields, robotic interfaces need to be intuitive to the general population and able to quickly interpret minimal user input from multiple modalities in directing autonomous teammates toward key locations for information-based tasking. This work presents a flexible multimodal algorithmic and visual interface that enables dynamic reprogramming of autonomous planning algorithms, focusing on the use of uncrewed aerial systems engaged in outdoor search and rescue. The Responsive Interface for iNtuitive Aircraft Operation (RINAO) leverages known geographic database information, such as trail networks, in conjunction with a variable set of user-defined features, such as search areas and landmarks, to efficiently infer a mission-specific, uncertainty-aware geospatial interest distribution that informs optimal planning algorithms through reward shaping. The approach is validated using 10 experts in public safety with 13.5 years of median operational experience. Results of this user evaluation show that the system enables effective and efficient alignment of geospatial interest and above-average usability. Evaluating the system’s performance against an inverse reinforcement learning (IRL) baseline, we find that our approach meets or exceeds the baseline’s value alignment while performing inference in substantially less time and with less user input. These results demonstrate that multimodal preference inference can enable rapid and intuitive mission specification for human—robot teams operating in time-critical environments.

## 1 Introduction

Robots engaged in dynamic, uncertain environments must be carefully directed by their human operators to be effective. Whether they are being deployed for public safety (Ray et al., 2022b), storm chasing (Frew et al., 2020), planetary exploration (Arora et al., 2019), undersea reconnaissance (Jamieson et al., 2020), or military operations (Bradshaw et al., 2013), robots must be tasked to be in the right place at the right time to provide a unique perspective or collect scientific data. Based on the situation at hand, an operator often has specific geospatial preferences for information-based tasking due to their physical geography or relative location. For example, a scientist may want their Mars rover to sample a unique rock found in an abnormal location, or a soldier may want their aircraft to patrol a specific segment of highway.

Implementing autonomous robots in these situations presents obvious benefits as they require less supervision from their operator(s) and offer them more freedom to engage with the environment, communicate, and collaborate with their human teammates. However, safe, reliable, and trustworthy autonomous systems additionally require a high degree of human control to help the system engage within the task’s context. In addition to providing a high degree of automation, an ideal system also provides operators with a high degree of control based on principles defined by Shneiderman (2022). This type of human-centered approach allows operators to shape the autonomous behavior in fulfilling the task through a collaborative but supervised engagement.

The use of uncrewed aerial systems (UASs) in public safety represents a widespread use of human–robot teaming in daily operations, as detailed by Ray et al. (2022b), and therefore presents an attractive medium for testing new methods of interacting with autonomous systems. This work is motivated by the challenges of deploying UASs in backcountry wilderness search and rescue operations; specifically, we consider representative scenarios in the search for people in a mountainous environment. UAS teams responding to this mission must account for the context of the particular incident to inform geographic tasking. This context can include the victim’s profile, geography of operations, prior and current weather conditions, locations of other teams, capabilities of their aircraft, and their specific mission tasking. All this information must be gathered from various modalities, including conversations, radio communications, historical databases, paper or electronic maps, weather stations, and other internet sources.

Although this context could theoretically be input into a black box generative model to output a mission plan, a competent operator would still need to review and approve the plan prior to engaging their aircraft. In any uncertain dynamic environment, it is unlikely such a generative model would be able to reliably act upon complex multi-modal information without supervision. This level of complexity can lead to brittle autonomous behavior, prone to failure when problems require nuanced context. Keeping the operator central to the system’s direction enables greater flexibility while empowering them with the responsibility to act upon their training and knowledge. However, ensuring that the operator can effectively direct the system requires careful alignment of human–robot mental models, especially with respect to mission goals. To warrant utilization in challenging situations, this alignment should be performed in a flexible and intuitive manner, ideally reflecting communication between human teammates.

**FIGURE 1:**
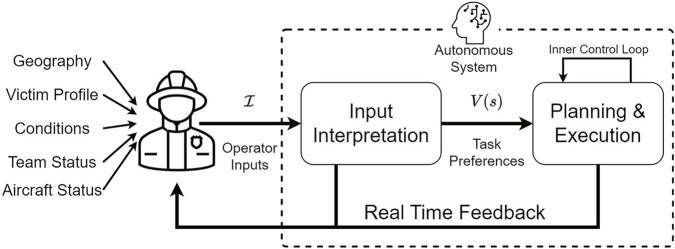
Incidents such as search and rescue require operators to fuse multiple sources of information to appropriately direct an aircraft. Our work aims to define and interpret a set of inputs that characterize an operator’s preferences over the planning and execution of an autonomous system.

While most approaches achieve effective direction from human preferences through comprehensive, mission-specific architectures (Burks, 2020; Jamieson et al., 2020; Arora et al., 2019; Scheutz et al., 2017), this work introduces a two-part architecture as defined in Figure 1. We provide a flexible method of interpreting the user’s inputs to understand mission constraints and, depending on the mission, leverage various methods to plan and act over the inferred goals. In dynamic and uncertain environments, the system needs to use minimal inputs to infer and align with the user on the relative importance of environmental features. Critically, this must include the incorporation of new, previously unknown, features, such as operating areas or regions of interest. This is addressed through a structured algorithmic foundation coupled with a flexible, multi-modal user interface that allows operators to dynamically add new features to the environment. Features are added using a sketch-based interface coupled with structured semantic language, such as “Go north of the neighborhood,” which probabilistically models locations’ likelihood of fitting that directive. Additional inputs include locations to visit or avoid and specific feature priorities. Our algorithm uses this limited operator-provided data and geographic database information, such as trail networks and tree canopy, to infer the mission importance of geographic and user-defined environmental features, which is captured using a spatial reward function. As different users may have unique perceptions and methods of communicating spatial intent, a guiding aim of this research is to present a relevant solution and evaluate whether operator and agent mental models can be aligned with respect to geospatial preferences. The resulting system is evaluated with 10 public safety experts in a realistic scenario taking place across 39 square kilometers (15 square miles) of wilderness to validate the overall system’s usability and algorithm value alignment. The approach is compared to a baseline that leverages inverse reinforcement learning (IRL), and the results show that the RINAO algorithm achieves similar or better value alignment with an order of magnitude less data and computation time. Subjects rate the interface as highly usable and easy to learn, although additional structured training would prove helpful prior to operational use.

In summary, this study presents the following contributions: 1) a flexible, multi-modal, and intuitive interface that can dynamically reprogram an autonomous agent’s direction in a data-driven environment; 2) an efficient method of aligning human and robot geospatial value; and 3) an expert-focused user evaluation that validates this approach in the context of using UASs in a search and rescue incident.

These contributions significantly extend prior work by Ray et al. (2024) through methodological improvements, a comprehensive software interface, and the application of a detailed value alignment metric. Specifically, our algorithm now includes a user accuracy parameter that considers possible user error and allows users to draw non-convex geometry when specifying custom features. A software interface has been developed that implements our algorithm in real time, allowing users to define their inputs and visualize its results. Finally, a validated metric from Sanneman and Shah (2023) serves as the basis for evaluating the algorithm’s capacity to align its estimate with the operator’s mental model.

## 2 Motivating problem

Although some architectures that enable comprehensive human–robot teaming in autonomous systems provide algorithms that must plan for the mission and gather human input, this can limit the complexity of the operator’s input (Jamieson et al., 2020) and efficiency of the planner (Burks et al., 2023). A concept of an autonomous system is shown in Figure 1, which divides the interpretation of the operator’s inputs, I , and the planning and execution over those inputs based on a spatial value function, V(s) . This allows the operator to define a mission using a complex set of I , which can then be interpreted in a manner that any number of motion or path planners may be able to understand and execute. A geospatial value V(s) , or state-based reward, that accounts for an operator’s preferences serves as an effective medium over which an autonomous system’s behavior can be constrained and reprogrammed. For example, if the baseline behavior of a search algorithm relies on a Markov decision process (MDP) (Kochenderfer et al., 2022), the reward function can be easily augmented with the operator’s reward so that $R\left(s,a\right)=R_{\text{baseline}}\left(s,a\right)+V_{\text{operator}}\left(s\right)$ . Other path planning algorithms, such as rapidly exploring random trees (LaValle, 1998), can also account for a cost value in their planning approach.

Specifically, let us assume that we have a landscape, which has been interpreted as a two-dimensional (2D) geographic map and then divided into a grid, G , with specific cells, g , at an arbitrary resolution. Each g contains an associated set of features captured in a vector, $\phi_{g}$ , that contains n mission-influencing static factors (such as waterways and trails) or dynamic factors (such as wind speed, team locations, and distance from the operator). The factors can be known a priori based on geographic information and also augmented by the user as different factors, or contexts, manifest themselves. The operator adds a set of inputs, I , which define the mission at hand. Based on these inputs, we seek to infer a preference-defining function f(I) . The function f(I) defines the spatial value function, V(s) . In this context, V(s)≜f(I) , where $f:R^{n}\mapsto R$ , converting multiple input dimensions into a single spatial value using a general parameter Θ :

$$f\left(\Theta ,\phi_{g}\right)=r_{g},$$ (1)

where $r_{1}>r_{2}$ implies that $g_{1}$ is a more valuable location for information-based tasking than $g_{2}$ . In wilderness search and rescue, this tasking can take the form of imaging a certain area using an RGB or thermal camera.

### 2.1 Grounding scenario

**FIGURE 2:**
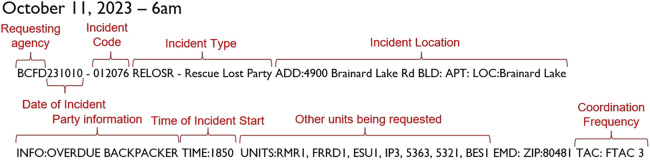
Scenario text page, reflecting a realistic backcountry search and rescue scenario.

While the interface is designed to support a broad range of uncertain and dynamic human–robot teaming applications, the motivating problem is intentionally grounded, without loss of generality, in a realistic backcountry search and rescue incident. The following scenario is drawn from the first author’s direct experience as a volunteer rescuer and reflects standard practices and agency protocols for emergency services in Boulder County, CO (Ray et al., 2022b). This scenario highlights key pieces of information that can drastically influence the execution of the search depending on the relative context. This scenario is referenced throughout the study and serves as the foundation for the user evaluation:

At 6 a.m. on October 11, our rescuer is woken by an alert and the text page shown in Figure 2. The first thing they notice is the incident type and location, which, in this case, is a search for an overdue backpacker in the vast Indian Peaks Wilderness area, starting from the Forest Service’s Brainard Lake Recreation Area. These details influence the gear that the rescuer carries, the incident’s jurisdiction, and the associated nature of the team organization. Seasoned rescuers also recognize that additional information will be needed, given that the recreation area has multiple trailheads and, therefore, multiple locations for the command post. The rescuer also notices that the incident date and time are from the previous night, which can mean that officials may have already started investigating leads and deem this a credible mission. They note the coordination frequency and additional resources that were also deployed, including the RMR mountain rescue team (Rocky Mountain Rescue), the FRRD dog teams (Front Range Rescue Dogs), and other command and support units. The combination of these details determines the available resources for the operation, which influences the nature of the individual tasks. For example, coordination on FTAC3 is not the primary coordination frequency. If the primary coordination frequency is occupied, a separate incident may be using that channel, which may limit the current availability of search teams, or suggest other issues with the communications infrastructure.

Upon arrival at the command post, the following additional information is received. The reporting party, who called in the incident, is the victim’s recent ex-girlfriend. Due to the breakup, this suggests a possible mental health condition and the associated tasking of examining more closely at cliffs in the event the victim has suicidal tendencies. The victim is reported to be a 23-year-old male in “OK” shape, who may have attempted to reach Pawnee Peak after an overnight. These details help define the subsequently large search area covering hundreds of square miles, as shown in Figure 5. The rescuer is also made aware that the victim’s vehicle was noticed since October 9 on the east side of Brainard Lake, which is an uncommon access point for the stated goal of Pawnee Peak. The weather yesterday had unexpected snow and high winds, which means that our victim may have taken shelter in an area with Western protection to account for the prevailing winds in the area. Initial reports are that two recent campsites were found in the Northern and Southern valleys. As an additional team is searching the Northern Valley, the rescuer is tasked with the Southern Valley to perform a hasty search, characterized by a rapid sweep along primary linear features, such as trails.

The summary of these details reflects the complexity of public safety incidents, with substantial implied information and relative uncertainty arising from correlating factors. Considerable differences across incidents mean that historical data cannot be solely relied upon to directly infer search assignments, and a “fully autonomous” teammate that does not collaborate effectively would be a burden to the incident. Instead, autonomous systems are applied using a human-centered approach from Shneiderman (2022), in which the operator retains a high level of autonomy while maintaining an appropriately strong degree of human control, enabling the behavior of the system to be tailored to the context at hand.

## 3 Related work

Experts engaged in field operations leverage years of training, operational experience, local knowledge, and mission information to form their mental model, which they then use to inform tasks to subordinate human or robotic teammates (Rouse and Morris, 1986). Autonomous systems operating in such dynamic and uncertain environments require a consistent method of communicating information from their underlying mental model, both within and across diverse platforms, to improve coordination as systems scale. Tabrez et al. (2020) explained how robots can interpret mental models in multiple ways, and robotic teams that use shared mental models were shown by Gervits et al. (2020) to improve their performance. However, many mental model frameworks rely on static, predefined architectures shared by all actors (Scheutz et al., 2017; Albrecht and Stone, 2018). The use of cross-training, where humans and robots switch roles to learn a collaborative task, was shown by Nikolaidis and Shah (2013) to effectively align mental models but is not a suitable approach for our application, where robots have different capabilities than their human teammates. In uncertain and dynamic applications of human–robot teaming with potentially multiple heterogeneous platforms, maintaining user interface interoperability with the underlying autonomy ensures consistent performance. By estimating an operator’s geospatial preferences in the form of a value function, V(s) , that is easily interpreted by multifarious autonomous planning approaches, this research addresses a more flexible “plug-and-play” approach that places greater onus on the operator for immediate direction while accommodating diverse underlying autonomous architectures.

In practice, autonomous robots have been successfully directed by their operators using other human-autonomy interaction methods. The approach detailed by Jamieson et al. (2020) uses a POMDP to mix the operator’s input interpretation with the planning execution for robot search tasks, although their approach severely limits the complexity of operator inputs to binary feedback. As shown in Figure 1, the RINAO algorithm decouples the user interface and planning components to leverage the richness of human cognition, fusing multiple types of inputs to inform generalized value functions. Expert knowledge has been applied in Arora et al. (2019), which uses Bayesian priors on geological knowledge to effectively define operator preferences for planning and execution but lacks an intuitive interface for non-engineers to create and update preferences in real time. More complex, multi-robot direction is realized through sets of “plays,” as described by Miller (2014), where a mission is broken down into smaller tasks but additionally requires a homogeneous autonomy architecture. Although operators can modify each task individually, there is no systematic way to define operator preferences across a whole mission set.

The approach taken by the Responsive Interface for iNtuitive Aircraft Operation (RINAO) algorithm developed here closely resembles the problem of preference elicitation (PE), where a system is tasked with inferring a user’s preference over a set of options and providing recommendations. Previous work by Ray et al. (2022a) recommended potential locations to UAS operators based on features identified in their previously provided points of interest. This earlier work is significantly extended here to encompass a greater definition of V(s) through multiple I and accounting for geographic features and geospatial structured semantic references. Most PE techniques focus on estimating a user’s preference based on previous data, such as news articles or food recommendations, using contextual multi-armed bandits (Li et al., 2010) and Gaussian processes (Bonilla et al., 2010). However, applying this approach to dynamic and uncertain environments with a modular, user-defined feature vector makes it impractical to rely on prior incident or user training data. Similarly, the number and type of inputs provided by the operator are subject to change throughout an interaction, which complicates the standardized inputs required by support vector machine or neural network methods for active learning-based single-user PE (Desmedt et al., 2021).

IRL problems resemble this problem in that the objective is to probabilistically model a user’s reward function. However, whereas IRL relies on observed expected behavior, such as reference trajectories, to directly infer reward functions (Arora and Doshi, 2021), this approach infers a geospatial reward distribution by fusing multi-modal inputs and readily available geospatial database information. Operators may not provide trajectories that optimally account for aircraft performance. Therefore, while operators could draw multiple reference trajectories to inform an IRL solution, we argue that learning low-level trajectory information is not as efficient as using a richer set of inputs to understand higher-level preferences in uncertain and dynamic human–robot teaming applications. Applying the learned distribution on top of a planner allows its baseline performance to be augmented by end-user expertise, addressing the challenges found with rewards designed by engineers (Hadfield-Menell et al., 2017; Booth et al., 2023). As IRL methods often learn a specific feature-weighting vector during the evaluation process, similar to our approach, a standard IRL method is applied (Abbeel and Ng, 2004; Kochenderfer et al., 2022) as a baseline for comparison in Section 5.

## 4 Methodology

**FIGURE 3:**
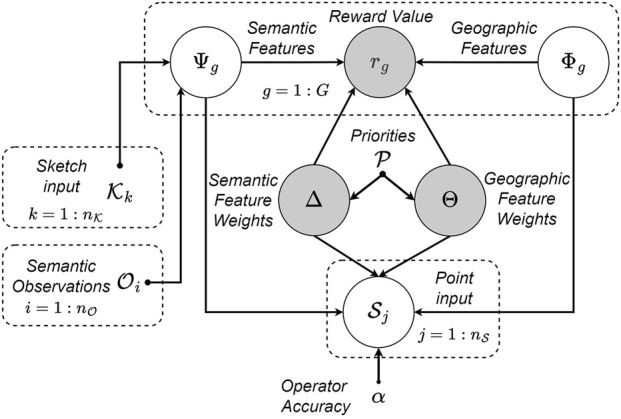
Graphical plate model of the system’s supporting algorithm. Grey random variables are inferred, while white variables are known; dash-boxed elements denote repeated substructures.

**FIGURE 4:**
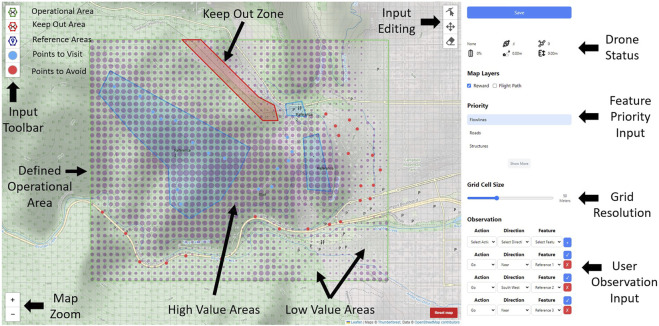
Developed user interface, which allows dynamic reprogramming of autonomous behavior through an intuitive web-based GUI and the supporting algorithmic framework.

The RINAO allows users to define a mission for an autonomous agent in a large-scale environment. The interface is defined by an intuitive UI, shown in Figure 4, that allows users to add inputs and new features to the environment, and the supporting probabilistic inference algorithm, implemented using the probabilistic graphical model shown in Figure 3, that uses said inputs to efficiently infer a feature weight vector and estimate geospatial reward values. Previous contributions (Ray et al., 2024) are expanded with the addition of a user accuracy parameter and support for users to define new environmental features using non-convex polygons.

### 4.1 Algorithmic approach

RINAO’s algorithmic approach for inferring geospatial reward values fuses multiple modalities of operator input and, therefore, provides the backbone of the user interface by enabling opportunistic and intuitive inputs in a manner that reflects communication between teammates. Teams involved in a search mission will first align on the critical features in the environment that will direct their search methods, which should be reflected in the direction of autonomous systems for information-based tasking. The features in the environment are defined as follows:

- Static geographic $\Rightarrow \Phi_{g}\in R^{n_{\Phi }}$ , corresponding to fixed landmarks including roads, trails, structures, flow lines, water bodies, and tree canopy.
- Variable user added $\Rightarrow \Psi_{g}\in R^{n_{O}}$ , corresponding to geospatial relations such as a cell g being inside or north of an operational area.

Based on these features and the set of inputs, I , RINAO infers a mission-specific feature weighting as the Θ parameter in Equation 1. Equation 1 is defined as a linear-weighting function expanded with the variable set of semantic features $\Psi_{g}$ and their corresponding weights, Δ , augmenting the static features, $\Phi_{g}$ , and weights, θ ,

$$r_{g}=\theta^{T}\Phi_{g}+\Delta^{T}\Psi_{g},\;\forall \;g\in G.$$ (2)

The corresponding mission weights are inferred, and user-defined features are created based on the inputs provided to the operator through our user interface.

The algorithm supports a set of three specific inputs, I , namely, reference points, S , feature priorities, P , and semantic geospatial observations, O . A geospatial observation, O , represents a structured semantic statement built from a qualifier (“Go” or “Don’t Go”), a choice of a predefined set of geospatial reference labels (“inside,” “near,” “north,” “southeast,” etc.), and an associated user-defined map-referenced sketch, K , which can represent a landmark or operational segment. Each sketch, $K_{k}$ where $k=1:n_{K}$ , is opportunistically defined by the user as a $R^{2}$ polygon projected onto G and given an associated label. Put together, the operator may direct an aircraft with the O input of “Go West of the Field,” which then increases the dimension of Ψ and Δ . Defining a feature priority, P , involves the selection of a feature label, either from one of the $n_{\Phi }$ static geographic features or one of the $n_{K}$ provided sketches, which, in turn, prioritizes observations referencing that feature within the inference process. Finally, the operator may add a set of $n_{S}$ reference points, projected onto G , which include locations to visit $\left(S_{j}=1\right)$ or avoid $\left(S_{j}=0\right)$ where $j=1:n_{S}$ .

Any of these I can be provided and modified throughout the mission as the operator deems necessary to shape the resulting reward. The provided set of I , which may or may not include sets of O , P , or S are fused as requested by the operator to infer and display the resulting value distribution, $r_{G}$ . The RINAO algorithm’s approach for inferring weights relies on operators to redefine and shape the mission given new information that is available to them. Mission requirements can change at a moment’s notice, for example, to support a ground team in scouring a difficult-to-access area, so RINAO does not inherently save operator geospatial preference information between input fusion instances.

While the scope of this work focuses on single events of interest, future work can explore how hyperparameters and user information can be saved and used over time. Fully Bayesian hierarchical methods, such as those presented by Ahmed et al. (2015), demonstrate one example of how sets of data from multiple users can be used to improve the resulting estimate. In the RINAO application, the interaction would be more effective if it learned directly from each individual user rather than from a wide body of experts. Learning from multiple interactions with a single user allows for a more personalized algorithm that adapts to how each user thinks as they may have unique interpretations of what constitutes “far” or “near.”

#### 4.1.1 Joint distribution model

The set of I are fused based on the probabilistic graphical model shown in Figure 3, which relates the inputs and associated features in white to the unknown random variables, θ , Δ , and $r_{g}$ , shown in gray. The set of static features Φ and user-provided features, Ψ , are first defined, followed by the inference method.

To define Φ , geographic features can be extracted from publicly available city, county, state, and national datasets. A geographical information system software program, such as ArcGIS, integrates a selected set of information, which is chosen to contain roads, trails, structures, flow lines, water bodies, and tree canopy. Flow lines are landscape features that channel water including dells, streams, creeks, and rivers. Additional information on the biome and infrastructure is readily available and could be included as necessary. A set of $\Phi_{g}$ should be defined that accurately maps an operator’s perspective of their respective value, which includes each g ’s proximity to a relevant feature. Therefore, for each g with a given resolution, the distance, d , to each of the closest respective features informs an adjacency metric, which is calculated using an exponential decay as $\Phi_{i,g}=\exp\left(\frac{-d}{\text{resolution}}\right)$ . All of the geoprocessing can be performed offline and saved into an accessible, resolution-specific database.

Each user-defined feature Ψ is created by a user’s observation input O . This is defined in prior work (Ahmed et al., 2013; Burks et al., 2023; Sweet and Ahmed, 2016; Burks, 2020), which probabilistically relates a geospatial semantic label to a given sketch, $K_{k}$ . Each K is initially provided as a series of vertices in $R^{2}$ . The available semantic labels include a comprehensive set of canonical bearing labels {“N,” “NE,” “E,” “SE,” “S,” “SW,” “W,” “NW”} and discrete ranges {“inside,” “near,” “outside”}. Modeling these labels with respect to a given sketch leverages the softmax function, which is effective for discrete-to-continuous mappings and overall pattern recognition (Bishop, 2006). Given a particular grid location g , with position $x_{g}\in R^{2}$ , Equation 3 approximates the likelihood of the semantic label:

$$p\left(class=i|x_{g}\right)=\frac{e^{w_{i}^{T}x_{g}+b_{i}}}{\sum_{j=1}^{K}e^{w_{j}^{T}x_{g}+b_{j}}},$$ (3)

where each class contains a set of parameters $w\in R^{2}$ and $b\in R^{1}$ , which are defined through algebraic manipulation to constrain their boundaries along the sketch border as presented by Sweet and Ahmed (2016) and Ahmed (2018). Once defined, Burks (2020) developed a Monte Carlo approximation to correlate the softmax classes with specific semantic labels, resulting in p(label|class) . Therefore, Equation 4 defines each $\Phi_{i}$ as p(label|g) , which is the probability of the given grid point being represented by a certain label:

$$\Psi_{i,g}=p\left(label|g\right)=\underset{\text{class}}{\sum }p\left(label|class\right)p\left(class|g\right).$$ (4)

We expand upon the approach discussed in Ray et al. (2024), which only allowed users to draw convex polygons with six vertices, by enabling the input of more complex, non-convex polygons with up to twenty vertices. This addition is important as larger features in the environment, such as neighborhoods, meadows, or canyons, are rarely convex, and accurately delineating and defining their relevance requires handling non-convex shapes. A non-convex sketch, K , is first broken down into a set of decomposed convex polygons, $\kappa_{i}\in K$ where $i=1:n_{\kappa }$ , based on the method described by Fernández et al. (2008). For each subpolygon, κ , the previously discussed softmax parameter, algebraic manipulation, and compass label approximations define p(label|class,κ) . Equation 5 defines the subsequent approximation where the maximum *a posteriori* (MAP) value over all subpolygons is applied as an efficient approximation resulting in the updated overall feature value, $\Psi_{i,g}$ :

$$\Psi_{i,g}=\max_{\kappa }\left\{p\left(label|g,\kappa \right)\right\}.$$ (5)

While the sets P and O help define the estimates and add features to the environment, the observable set of S critically ties the known feature components Φ and Ψ to their respective unknown weightings θ and Δ . It is assumed that each g must reach a specific threshold of an operator’s optimal positive or negative preference for it to be provided as a reference. The distribution $p\left(S_{g}|\theta ,\Delta ,\Phi_{g},\Psi_{g}\right)$ is modeled as a logistic function in Equations 6 and 7 with $r_{g}$ defined as in Equation 2.

$$p\left(S_{g}=1|\theta ,\Delta ,\Phi_{g},\Psi_{g}\right)=\frac{\exp\left(r_{g}\right)}{1+\exp\left(r_{g}\right)},$$ (6)

$$p\left(S_{g}=0|\theta ,\Delta ,\Phi_{g},\Psi_{g}\right)=\frac{1}{1+\exp\left(r_{g}\right)}.$$ (7)

Given that users often make mistakes or may account for unmodeled features in the environment, a predefined accuracy parameter is applied. α , this allows for some probability (1−α) that if the operator specified a location to visit, they may have actually intended to avoid that location. The addition of the accuracy parameter therefore respectively expands the definitions of Equations 6, 7 into Equations 8, 9.

$$\begin{array}{ll} & p\left(S_{g}=1|\theta ,\Delta ,\Phi_{g},\Psi_{g},\alpha \right)=\alpha \;p\left(S_{g}=1|\theta ,\Delta ,\Phi_{g},\Psi_{g}\right)\\ & \;+\left(1-\alpha \right)\;p\left(S_{g}=0|\theta ,\Delta ,\Phi_{g},\Psi_{g}\right), \end{array}$$ (8)

$$\begin{array}{ll} & p\left(S_{g}=0|\theta ,\Delta ,\Phi_{g},\Psi_{g},\alpha \right)=\left(1-\alpha \right)\;p\left(S_{g}=1|\theta ,\Delta ,\Phi_{g},\Psi_{g}\right)\\ & \;+\alpha \;p\left(S_{g}=0|\theta ,\Delta ,\Phi_{g},\Psi_{g}\right). \end{array}$$ (9)

Having defined the associated components, definitions for θ and Δ are now presented. It is assumed that θ and Δ are unbounded in the continuous domain and model priors for each of these variables as a multivariate Gaussian with respective means $\mu_{\theta }$ and $\mu_{\Delta }$ and co-variances $\Sigma_{\theta }$ and $\Sigma_{\Delta }$ . As the geographic features are considered fixed, θ also has a fixed dimension of $R^{n_{\Phi }}$ . The user-defined features vary depending on the number of observations, and therefore, Ψ has a dimensionality of $R^{n_{O}}$ . While $\mu_{\Phi }$ is initialized to a vector of zeros as user O are provided, $\mu_{\Psi }$ is initialized with a positive term, which in practice was set to a value of 1.5. Initializing with a positive value reflects the operator’s immediate interest in this feature. However, if a user provided a set of priorities, P , this acts as a prior on components of Φ and Ψ by adding a positive bias to the initialization of the associated feature weight’s $\mu_{i}$ and a reduced value of $\sigma_{i}$ . This input, therefore, allows the user to rapidly define features of interest that may otherwise have to be inferred solely through the input of S . Given these definitions, an a priori (pre-fusion) MMSE or MAP estimate can be defined for a given g ’s reward, ${\hat{r}}_{g}$ , and associated variance, $var\left({\hat{r}}_{g}\right)$ , as

$${\hat{r}}_{g}=\mu_{\theta }^{T}\Phi_{g}+\mu_{\Delta }^{T}\Psi_{g},$$ (10)

$$var\left({\hat{r}}_{g}\right)=\Phi_{g}^{T}\Sigma_{\theta }\Phi_{g}+\Psi_{g}^{T}\Sigma_{\Delta }\Psi_{g}.$$ (11)

#### 4.1.2 Model inference for value estimation

The graphical model in Figure 3 shows us how to calculate the overall joint probability p(r,Ψ,Φ,θ,Δ,K,P,O,S) . As the objective is to approximate V(s) through the reward proxy $r_{g}$ for all g∈G , $\hat{r}$ is inferred as the expected value of p(r|Ψ,Φ,θ,Δ,K,P,O,S) , which simplifies to p(r|θ,Δ,Ψ,Φ) based on conditional independence. A variety of methods can be used to perform inference via the graphical model, including Gibbs sampling (Ahmed et al., 2015), variational Bayes (Ahmed et al., 2013), or the Laplace approximation (Wakayama and Ahmed, 2023), as illustrated by Bishop (2006).

From the chain rule, follows Equation 12,

$$\begin{array}{ll} p\left(r,\theta ,\Delta |\right. & \left.\Psi ,\Phi ,K,P,O,S\right)\\ & =p\left(r|\theta ,\Delta ,\Psi ,\Phi ,K,P,O,S\right)p\left(\theta ,\Delta |\Psi ,\Phi ,K,P,O,S\right)\\ & =p\left(r|\theta ,\Delta ,\Psi ,\Phi \right)p\left(\theta ,\Delta |\Psi ,\Phi ,P,S\right), \end{array}$$ (12)

where the second equality follows from the conditional independence properties of the graphical model. From this, the posterior expected reward $E\left[r_{g_{i,j}}\right]$ can be approximated according to a slight modification of Equation 10 if only the joint posterior expected values and variances for (θ,Δ) are considered using the second factor in the RHS of Equation 12.

Thus, the focus is on approximating this second posterior factor p(θ,Δ|Φ,Ψ,P,S) . The inference must be flexible to a changing feature set and simple enough to be run in real-time. The Laplace approximation is well suited for these requirements. From Bayes’ rule, the posterior factor is proportional to

$$p\left(\theta ,\Delta |S,\Phi ,\Psi ,P\right)\propto p\left(\theta ,\Delta |P\right)p\left(S|\Phi ,\Psi ,\theta ,\Delta \right).$$ (13)

Since each $S_{i}$ is conditionally independent of the other,

$$p\left(\theta ,\Delta |P\right)p\left(S|\Phi ,\Psi ,\theta ,\Delta \right)=p\left(\theta ,\Delta |P\right)\overset{K}{\underset{g=1}{\prod }}p\left(S_{g}|\Phi_{g},\Psi_{g},\theta ,\Delta \right).$$ (14)

With p(θ,Δ|P) modeled using Gaussian priors and $p\left(S_{g}|\Phi_{g},\Psi_{g},\theta ,\Delta \right)$ modeled as logistic functions, the LHS of Equation 13 is not analytically tractable. The Laplace approximation approximates the RHS of Equation 14 and the normalizing constant C for Equation 13, thereby permitting a Gaussian approximation of $p\left(\theta ,\Delta |S_{1\ldots k},\Phi ,\Psi ,P\right)$ . Specifically, the posterior is approximated by fitting a Gaussian distribution over f(θ,Δ) , where the mean is equal to the MAP estimate $\left(\theta^{*},\Delta^{*}\right)$ (obtained via quasi-Newton optimization on log⁡f(θ,Δ) ) and the covariance matrix is the inverse of the Hessian A=H[log⁡f(θ,Δ)] , as defined by Bishop (2006), such that Equation 15 represents a relevant approximation where

$$f\left(\theta ,\Delta \right)=p\left(\theta ,\Delta |P\right)p\left(S|\theta ,\Delta ,\Phi ,\Psi \right)\cdot C\approx N\left(\left[\begin{array}{c} \theta^{*}\\ \Delta^{*} \end{array}\right],A^{-1}\right).$$ (15)

The computational demand for inference is driven by inversion of A , giving worst case complexity of $O\left({\left(n_{\Phi }+n_{O}\right)}^{3}\right)$ .

The execution of the algorithm requires defining various hyperparameters. These parameters were manually tuned through heuristic optimization over the data collected in Ray et al. (2024) and additional datasets collected in pilot evaluations. For our implementation, accuracy was defined as α=0.9 , which assumes a generally accurate user but leaves room for error. Each geographic feature was set with a prior of p(θ)=N(0.0,10.0) , which provides a zero bias and large uncertainty for all available static features. Each semantic feature was initialized with a prior of p(θ)=N(1.5,6.0) as this biases the provided input feature in a positive manner with a reduced uncertainty. A prioritized feature, either Δ or θ , received a highly biased prior of N(6.0,3.0) .

### 4.2 Interactive user interface

A graphical user interface is implemented as a JavaScript web-based application, as shown in Figure 4, where users can define their mission inputs. At a high level, the operator is presented with the map area, drawing options, and the sidebar where additional inputs can be specified. Starting with the map, users can visualize their area using a topographical map designed for outdoor recreation.^1^ The top left of the map contains the primary drawing tools. The top green button allows users to draw a polygon around the operational area, specifying the locations where the mission may take place. If the operational area is not explicitly added by the user, it is defined as the smallest rectangle that fits all the provided inputs. In the user study discussed subsequently, this area is predefined for all users. The red hexagon allows the definition of any no-fly areas, which automatically implements a prioritized “Go outside” observation on the draw area. Finally, the blue hexagon is what allows users to define reference areas, K , which can then be referenced with observations. Below those buttons are the blue points for defining specific locations to visit (S=1) and red points to indicate locations to avoid (S=0) . A polygon can be renamed by tapping on it, and its shape can be modified using the tools on the upper right of the interface. Users also have zoom buttons on the lower left and a reset option on the lower right.

The sidebar is located on the right of the interface. Starting at the top, the “save” button stores the inputs, performs the algorithmic inference, and displays the inferred reward values. These are shown as transparent magenta circles, where a bigger circle corresponds to a higher inferred value. Although additional detail could be displayed, such as estimate uncertainty, this approach aimed to balance operator workload and ease of use by limiting the level of detail provided, a further investigation of which is provided by Wang and Lau (2023). Below the save button, users have some basic flight telemetry that can be used to monitor aircraft status. Feature priorities can be defined by selecting one of several pre-defined geographic features or additional reference sketches as they are added by the user. Below the priority input, users can choose the level of resolution on the grid, which defines the scale at which geospatial inference is performed. Finally, users have the observation area, where one can select an action such as “Go” or “Don’t Go,” a direction, and the specific reference area. Once these have all been chosen, clicking the “+” sign adds the observation. A provided observation can be modified by changing its selection and selecting the check mark.

## 5 Expert user validation

The RINAO algorithm aims to provide a flexible interface for various users to define their spatial intent for information-based tasking. Our method of value alignment is validated against a relevant baseline through a limited user evaluation with experts (n=10) in the field of public safety. The maximum margin IRL algorithm by Abbeel and Ng (2004) is implemented as an additional method for inferring a feature-weighting vector and comparing the resulting value alignment with our own approach. The RINAO and IRL algorithms both have access to the same set of environmental features but differ in their interpretation of user interest, with RINAO relying on specific locations of interest or disinterest and IRL relying on trajectory data. Users also rate the system using a usability metric and provide general feedback on the interface and use of the system.

The motivation in performing this systematic user experience evaluation is to gather feedback on this modality of input and validate our method of alignment with a non-generalized, limited group of users. Through this study, two research questions relating to the performance of our approach are assessed:

1. Does the RINAO algorithm significantly align with the participant’s reward mental model compared to an inverse reinforcement learning baseline?
2. Does the RINAO algorithm enable more efficient inference of the operator’s reward model compared to an inverse reinforcement learning baseline in terms of computation speed and data requirements?

### 5.1 Baseline algorithm

A baseline algorithm is implemented to learn the operator’s geospatial value model based upon the textbook maximum margin inverse einforcement learning approach from Abbeel and Ng (2004) as presented by Kochenderfer et al. (2022). A key requirement of any IRL method is the definition of a grounding MDP for which the algorithm attempts to learn the resulting reward function R(s,a) . An MDP is a tuple (S,A,T,R,γ) , where S and A are finite sets of states and actions, respectively, T:S×A×S→[0,1] is the transition probability function, R:S×A→R is the immediate reward function, and γ∈[0,1] is the discount factor (Kochenderfer et al., 2022). The value function for a given state V(s) is defined in Equation 16 as the infinite horizon expected total reward:

$$V\left(s\right)=E\left[\overset{\infty }{\underset{t=1}{\sum }}\gamma^{t-1}r_{t}\right].$$ (16)

To this end, a basic MDP is defined that aims to collect as much user-defined V(s) or “reward” as possible across the overall grid, G , mirroring the behavior of the classic Pac-Man video game.

#### 5.1.1 Pac-Man MDP

This MDP is a purely operator-driven approach in that its sole purpose is to move about the grid and collect rewards based solely on the inferred geospatial value. The elements of the MDP are defined as follows:

S: {robot position ⇒g∈G , robot position history $\Rightarrow \left\{g\in G\right\}\in R^{H\times 2}$ }

A: Move in one of the following directions on the grid: north, northeast, east, southeast, south, southwest, west, and northwest.

R:

$$\left\{\begin{array}{cc} \text{if Position}\notin History & r=r_{g}\\ else & r=0 \end{array}\right..$$

T: The robot transitions to the intended cell with probability α and otherwise transitions uniformly to other directions. When the robot moves, its previous grid-world position is added to the state within the position history.

Discount factor (γ) : This is set to 0.95 to reflect more immediate prioritization of the flight path.

Termination: The MDP reaches a terminal state when the length of the history is equal to a predefined constant, $H_{\max}$ . In practice, this is set to 50.

#### 5.1.2 Solver

As the MDP formulation results in a very large state space $\left(|S|=H_{\max}\times {\left(n\times m\right)}^{2}\times 2^{\left(n\times m\right)}\right)$ , we solve the problem using an online solver. For our purposes, Monte Carlo Tree Search (MCTS), as defined by Coulom (2006) and implemented in the Julia POMDPs.jl library by Egorov et al. (2017), proves to be adequate. An epsilon-greedy rollout policy (ϵ=0.3) is used alongside an exploration constant of 1, depth of 50, and 1000 iterations.

#### 5.1.3 Maximum margin inverse reinforcement learning

Our baseline method for learning the resulting feature weights, Θ , is the classic maximum margin IRL algorithm by Abbeel and Ng (2004). This method has access to the additional semantically defined features, $\Psi_{g}$ , at each location but differs from RINAO in that the data collected to learn the feature weights is provided in terms of a set of reference trajectories. Assuming that the expert is maximizing features in the environment with specific frequencies, this IRL approach trains the underlying MDP to replicate the provided pattern of activation. Given a set of expert-provided trajectories, the optimization process is used as specified in Abbeel and Ng (2004) and implemented in Kochenderfer et al. (2022). As the operational area is approximately $38.5{km}^{2}\;\left(15m^{2}\right)$ , subjects can draw multiple trajectories within the environment starting from four defined launch locations within the operational area. The optimization process is then run for the four different positions, and the resulting average is considered the mission value of Θ .

### 5.2 Evaluation methods

The RINAO algorithm and interface are evaluated using a combination of metrics to assess value alignment, usability, inference computation time, and data requirements. The primary reward alignment metric leverages the four-part approach developed by Sanneman and Shah (2023), hereafter referred to as the Sanneman approach, which decomposes overall reward and value alignment into feature and policy components. After subjects complete their data input on the interface, they begin the *Free Response* section, where they write down a set of features, $F_{H}^{fr}$ , that are important to their decision-making and subsequently rank them in order of importance. These rankings create a set of pairwise comparisons of the absolute values of their relative weights, $W_{H}^{fr}$ (e.g., $\left|w_{A}|>|w_{B}\right|$ , where $w_{i}$ is the weight of feature i ). The evaluated algorithm infers a corresponding set of relevant features, $F_{R}^{fr}$ , and rankings leading to $W_{R}^{fr}$ . Equation 17 is used to determine a normalized value:

$$FR=\frac{\left(F_{H}^{fr}\cup W_{H}^{fr}\right)\cap \left(F_{R}^{fr}\cup W_{R}^{fr}\right)}{\left(F_{H}^{fr}\cup W_{H}^{fr}\right)\cup \left(F_{R}^{fr}\cup W_{R}^{fr}\right)}.$$ (17)

For example, the user may only value two features (trails and streams), with trails being a higher priority, while the algorithm determined that three features are valuable (trails, streams, and woods), with woods being the least valuable and trails being the most valuable. Here, $F_{H}^{fr}=\left[Trails,Streams\right]$ and $W_{H}^{fr}=\left[Trails>Streams\right]$ , while $F_{R}^{fr}=\left[Trails,Streams,Woods\right]$ and $W_{R}^{fr}=\left[Trails>Streams,Trails>Woods,Streams>Woods\right]$ . The two overlapping features, single overlapping ranking, and six distinct items lead to Equation 18:

$$FR=\frac{3}{6}=0.5.$$ (18)

In the next step, *Feature Selection and Ranking*, subjects select which specific decision-making features are important in the scenario and rank them in order of importance. For our study, this includes the six static geographic features and any additional subject-provided observations. Any feature not deemed important is left blank. These rankings are compared with the algorithm’s inference according to Equation 17.

**FIGURE 5:**
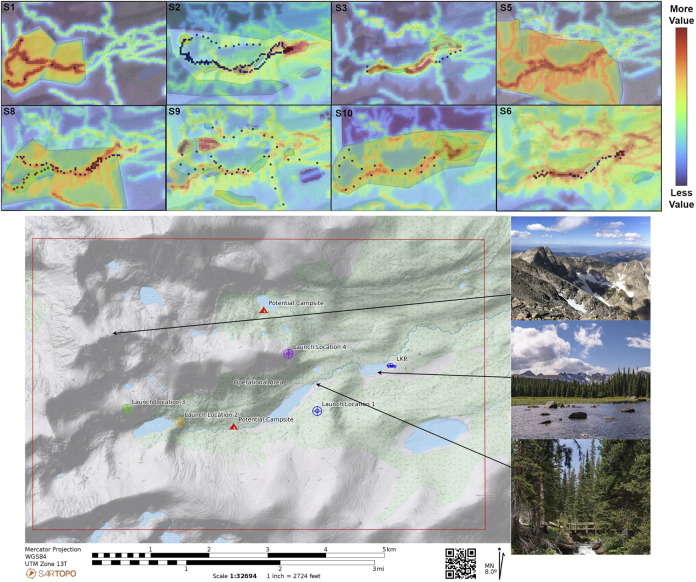
Results from RINAO showing various modalities of input leading to greater or lesser concentration of value across the operational area. The Brainard Lake area of operations is shown, with images highlighting the diversity of terrain.

Step three is the *Best Demonstration* section, which asks the subject to mark an ideal path that maps the aircraft’s trajectory to its furthest extent. Due to the large area covered in our scenario, subjects are asked to draw paths starting from four specific launch locations shown in Figure 5. Each of the resulting i trajectories, $\xi_{i}^{H}$ , is evaluated using the inferred reward function to generate an estimated discounted reward $R_{R}\left(\xi^{H}\right)$ . Using Equation 19, this is compared against the Pac-Man MDP’s evaluated optimal path, $\xi_{i}^{R}$ , and corresponding discounted reward, $R_{R}\left(\xi^{R}\right)$ , to determine a normalized best demonstration metric,

$$BD=1-\frac{R_{R}\left(\xi^{R}\right)-R_{R}\left(\xi^{H}\right)}{R_{R}\left(\xi^{R}\right)}.$$ (19)

The final step, *Preference Elicitation*, asks subjects to evaluate 12 pairs of previously designed trajectories starting at each of the four launch locations. Trajectories were designed with conflicting goals in mind, such as following a path or flowline while moving in opposing directions. The final metric is evaluated as the percent of overlap between the subject’s choice, $q_{H}$ , and the path deemed more optimal by the Pac-Man MDP, $q_{R}$ . Subjects could select option A, B, or be unsure. We considered the algorithm to be “unsure” if the resulting reward value was within 1% between the two trajectories. These choices are used in Equation 20 to define the PE metric as the percent of overlap in responses between the human and algorithm (i.e., recall),

$$PE=\frac{\left|q_{H}\cap q_{R}\right|}{q_{R}}.$$ (20)

Additionally, a location rating metric is used, which aims to directly evaluate the area-wide geospatial value error between the subject, $r_{g,\text{true}}$ , and the algorithm’s estimate, ${\hat{r}}_{g}$ . For 21 specific locations, subjects were asked to give an integer rating of −1 (avoid) to 2 (important to visit). This limited rating range aimed to limit decision fatigue and simplify the overall qualification task. The total error (e) is calculated by comparing the operator’s $\left\{-1,0,1,2\right\}$ ranking by partitioning $\hat{r}$ across G into quartiles and assigning the respective quantity of $\left\{-1,0,1,2\right\}$ . We additionally weight this difference using the algorithm’s uncertainty for that particular location, $var\left({\hat{r}}_{g_{i,j}}\right)$ , as defined previously. When applying the location rating metric to the baseline approach, Equation 21 uses the variance of the inferred weights as $var\left({\hat{r}}_{g}\right)$ , defined in Equation 11.

$$e=\overset{21}{\underset{g=1}{\sum }}\frac{{\left({\hat{r}}_{g}-r_{g,\text{true}}\right)}^{2}}{var\left({\hat{r}}_{g}\right)}.$$ (21)

The computation time and data requirements are used to evaluate the computational efficiency of the system. To estimate computation time, all software programs are written in the Julia computing language, and the Benchmark Tools software library^2^ is used. The RINAO inference and IRL algorithms are each run multiple times, and the average CPU time is reported to infer a feature weighting for each subject’s inputs. Data requirements for each algorithm are considered by evaluating the data requirements that each algorithm needs to perform inference. In this case, both algorithms use the sketch and semantic observation information, so the primary difference lies in the availability of point inputs. Therefore, the resulting data requirement metric is the percentage of additional points required to either form a trajectory or define key locations that should be visited or avoided.

The System Usability Scale (SUS) by Brooke (1995) is used to quantitatively evaluate the usability of the system. This metric provides a well-tested, subjective measure to understand subjects’ perception of the overall system. It consists of a series of 10 questions, each of which the subject rates on a Likert scale from 1 (strongly disagree) to 5 (strongly agree). The 10 scores are aggregated to generate a summary usability score from 0 to 100, where 100 indicates the highest possible usability. According to Sauro (2011), a SUS score of 68 or higher indicates above-average usability.

### 5.3 Procedure

Each subject evaluation sessions were conducted one-on-one between the participant and the researcher. The session started with a briefing of the research motivation and goals for the experiment. The researcher presented a high-level concept of operations describing how the specific system would integrate into current rescue operations. The available inputs were then described at a high level, and a few examples from previous work by Ray et al. (2024) were provided to show different ways of using the inputs. The researcher then started the RINAO user interface (UI) on a touch-based tablet and walked the subject through the various inputs and capabilities. The subject was allowed to “play” with the interface and try different inputs, which often led to longer conversations about how the system works. Once they were comfortable with the UI, the researcher provided them with the associated scenario information as described above. Subjects then added their inputs on the map, which had a predefined operational area and a resolution set to 100 m. After subjects were satisfied with the resulting reward map, they began the validation portion of the experiment. This first included the *Free Response* and *Feature Ranking* section using the associated worksheet. The subject’s *Best Demonstration* of a flight from each launch location, shown in Figure 5, was collected on a separate application. Subjects were then asked to draw additional reference trajectories on the map that they felt would adequately cover the area and satisfy their initial tasking for a hasty search. Finally, their *Preference Elicitation* and *Location Rating* inputs were collected before they filled out an online SUS questionnaire and demographics survey.

### 5.4 Demographics

**FIGURE 6:**
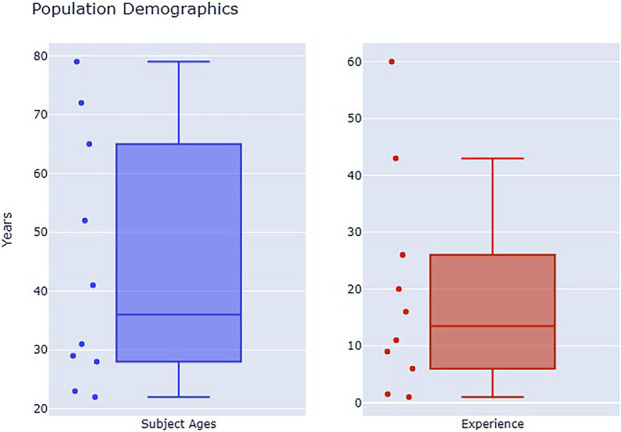
Demographics of evaluated expert users show a broad diversity of age and experience.

Ten diverse and experienced public safety operators tested the realized system. All subjects were required to have completed basic training and have additional qualifications as a Part 107 UAS pilot or have taken a course in search and rescue incident management. As shown in Figure 6, subjects were aged 22 to 79 years ( μ=44.2 , $\mu_{\frac{1}{2}}=36$ ) and had 1–60 years of experience in public safety ( μ=19.4 , $\mu_{\frac{1}{2}}=13.5$ ).

### 5.5 Results

A selection of users’ inputs is shown in Figure 5. Feedback from the 10 operators was generally positive, with participants expressing enthusiasm about the potential provided by the interface. These are reflected in various comments indicating that the system, “was a quantum leap in capability,” that “it is pretty badass,” and that “this is really cool.” More specifically, one subject mentioned that “having this capability lets me be more of a searcher than a pilot.” This attitude was often reflected in the fact that multiple subjects vocalized conflict regarding adding inputs that corresponded to basic search management principles or more specific flight planning. For example, subject 3 assumed an initial starting location and added waypoints to an area that they wanted the aircraft to explore more freely. Conversely, subject 9 marked various areas where lost persons would be attracted to and then added reference observations of those locations. An additional variation across subjects was in the quantity of inputs provided. Subject 2 added the largest number of observations (9), priorities (10), and points (190), leading to significant complexity in the resulting value map. On the other hand, subject 1 provided a single observation, two priorities, and six positive reference points while commenting that they would “rather do more detailed mission planning” for specific flights and launch locations. These comments and differing methods of using our interface highlight its flexibility in integrating varying quantities of user inputs, which could suit a diversity of resulting mission designs depending on the rest of the system’s configuration.

**FIGURE 7:**
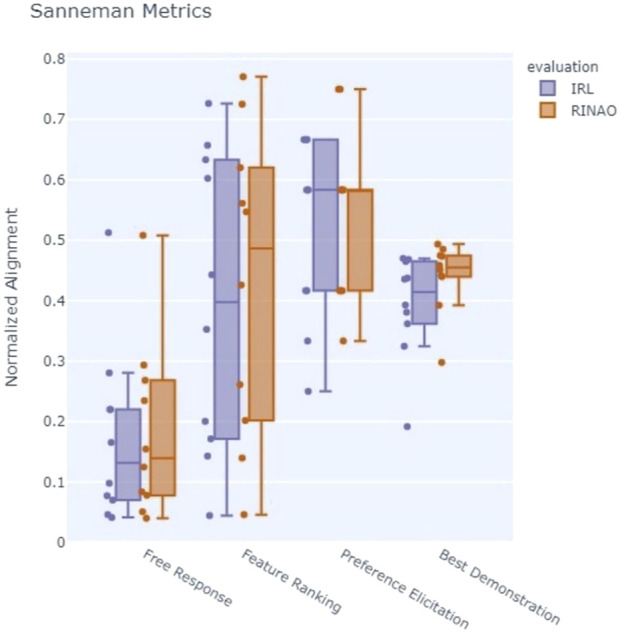
Results of the user study across Sanneman evaluative criteria, where higher values indicate better performance, show that our approach (RINAO) often meets or exceeds alignment compared to the IRL baseline.

**FIGURE 8:**
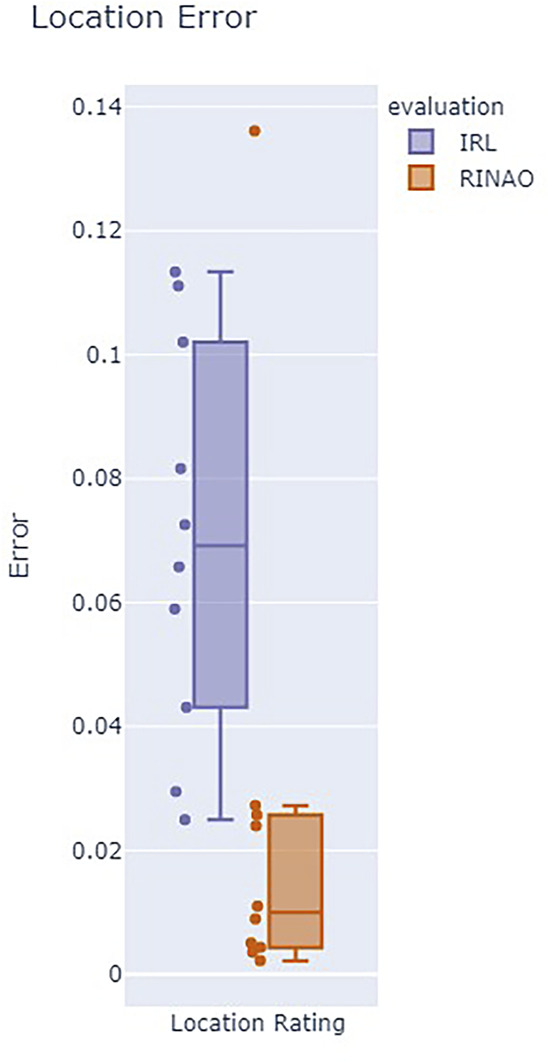
Results of the user study using the location error metric, where a lower value indicates a better performance, show that our approach (RINAO) performs significantly better.

Results for the value alignment metrics are shown in Figure 7, which shows the four components of the Sanneman approach, and Figure 8, which shows the location error rating. Formal statistical analysis is performed using a Wilcox signed-rank test, although the small sample size of experts limits the generalization of the results. The Feature Alignment evaluation metrics are first evaluated, which include the *Free Response* and *Feature Ranking* components. Considering the *Free Response* metric, whose ideal value is 1, both methods have rather poor alignment with the subject. However, they generally fall within the same range and share a similar mean $\left(\mu_{\text{RINAO}}=0.18,\;\mu_{\text{IRL}}=0.17\right)$ , although there is a significant difference between conditions (p=0.0098, d=0.85) . The overall low alignment value can be attributed to two key factors, including a generally low number of user-provided features (μ=6.3) and some subjects not fully understanding the question. For example, one subject marked that “Footprints” were considered a valuable feature in their decision-making despite this not being a specific map feature that could influence their geospatial interest. The most commonly considered feature that the system did not have the ability to account for was “Peaks” and “Ridges,” which suggests that additional topographical or elevation data would be a helpful feature to implement. Evaluating the *Feature Ranking* metric, the RINAO algorithm had a higher mean value than the baseline $\left(\mu_{\text{RINAO}}=0.43,\mu_{\text{IRL}}=0.39\right)$ , although the difference was not significant (p=0.16) . However, both the RINAO and IRL method have similar ranges of alignment and do not present a significant difference. The results of the *Feature Ranking* metric are substantially higher than the comparable *Free Response* results, suggesting that both RINAO and IRL can adequately match feature weightings, depending on the inputs provided to the operator and their perspective on how those inputs influence the resulting inference. However, these results correspond to the inputs from a single complex scenario over a wide area, and future work will attempt to evaluate alignment across multiple possible scenarios and a generalized population.

The Policy Alignment metrics are next evaluated, which include the *Preference Elicitation* and *Best Demonstration* components. In the *Preference Elicitation* evaluation, the RINAO algorithm generally presents a higher mean value compared to the baseline but does not have a significant difference $\left(\mu_{\text{RINAO}}=0.57,\;\mu_{\text{IRL}}=0.49,\;p=0.56\right)$ . The *Best Demonstration* evaluation shows that RINAO is higher than the baseline $\left(\mu_{\text{RINAO}}=0.45,\;\mu_{\text{IRL}}=0.38,\;p=0.065\right)$ . This suggests that RINAO can capture slightly more contextual nuance than the baseline approach. Notably, the baseline method performed worst on Subject 2’s inputs, which also included the highest number of trajectories (n=14) provided by any subject.

The final value alignment metric is *Location Rating*, which is not a part of the Sanneman reward alignment tests. This error metric, whose ideal value is 0, showed that our approach had a significant improvement compared with the baseline $\left(\mu_{\text{RINAO}}=0.025,\mu_{\text{IRL}}=0.062,\;p=0.02\right)$ . Specifically, we evaluated the comparison using a two sample t-test with unknown unequal standard deviation $\left(\sigma_{\text{RINAO}}=0.041,\sigma_{\text{IRL}}=0.029\right)$ and found that the result was significant (α<0.05) . This difference may result from the different approaches to inferring problem uncertainty as our method more directly accounts for uncertainty in specific feature weightings.

**FIGURE 9:**
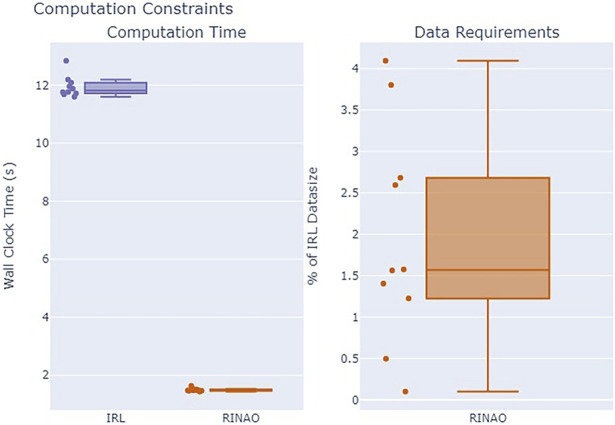
Inference efficiency for the evaluated algorithm compared to the IRL baseline.

Evaluating the inference methods between the two approaches, Figure 9 shows that RINAO is significantly faster and requires substantially less data. Computation was performed on a consumer-grade laptop, and both codebases are entirely written in Julia. The RINAO algorithm was an order of magnitude faster than the baseline $\left(\mu_{\text{RINAO}}=1.74s,\mu_{\text{IRL}}=13.97s\right)$ and used an average of 1.6% of the data required for the baseline solution. Since the IRL approach maintains access to the polygon information, the primary difference in data use arises from the amount of information provided by the full trajectories needed in IRL versus the specific points of interest that can optionally be provided by RINAO. These differences allow operators to communicate geospatial goals with less information, which can be rapidly acted upon by a computationally constrained system.

**FIGURE 10:**
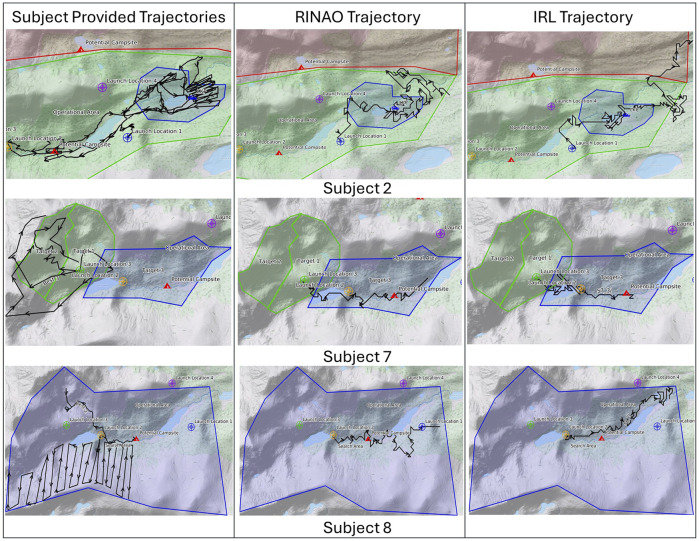
Selection of trajectories provided by subjects, generated by RINAO and IRL. Each subject’s sketches are overlaid on the resulting map, with red areas representing keep-out zones and blue areas representing prioritized regions.

The Pac-Man MDP plans over the resulting information in unique ways, as shown in Figure 10. This figure presents a selection of subject trajectories for particular launch locations, which vary considerably depending on the subject, along with the resulting optimal actions from the Pac-Man MDP applied over the RINAO and IRL results. Subject sketches are overlaid with red polygons to designate keep-out zones, and blue polygons are prioritized regions. Subject 2 provided four detailed trajectories from launch location 1. Acting over these inputs, the IRL trajectory violates the keep-out-zone in an effort to follow a trail, whereas the RINAO-augmented trajectory performs a more comprehensive exploration of valid trail systems while avoiding road areas. Subject 7 provided three trajectories from launch location 3, which explore the upper valley. In contrast, both RINAO and IRL methods lead the Pac-Man MDP toward the prioritized lower valley. In this case, the RINAO method successfully deprioritizes the body of water and focuses on the trail and flow lines but is generally similar. Subject 8 provides three trajectories from launch location 2, which explore nearby flow lines and perform a detailed grid search. While IRL focuses on the trail, RINAO executes a broader search that explores a nearby ravine (flow line).

**FIGURE 11:**
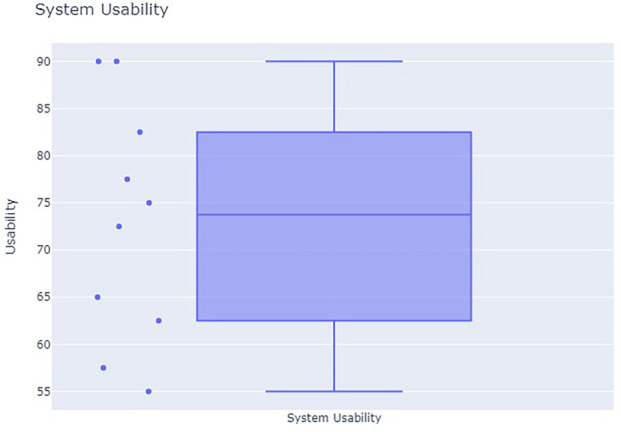
Results from the system usability scale.

Results from the SUS, shown in Figure 11, indicate that the RINAO algorithm is easy to use. The average score across subjects was 73, which is considered to be indicative of high usability in the reference literature (Sauro, 2011). The highest adapted component score (μ=3.4) was from question 4, “I think that I would need the support of a technical person to be able to use this system,” showing the approachable and intuitive nature of the visual interface. This result provides concrete validation of the effectiveness of the chosen user inputs, especially in how they reflect common patterns of communication between human teammates. The lowest-scored component (μ=2.4) was from question 9 “I felt very confident using the system,” reflecting a need for improved training and experience with the system. Despite the high level of subject experience, the novel concept of operations that RINAO provides, with respect to providing high-level direction versus low-level controls, will take time for users to familiarize themselves with. Conversely, the component with the highest variability in response was question 6, “I thought there was too much inconsistency in this system.” The current interface provides multiple ways of communicating similar information. For example, in a simple case where a user wants to focus on trails, they can either select “trails” as a priority or place multiple positive points on locations with trails. This was an intentional design choice as the use of the priorities can simplify the communication of important features without requiring significant user input. The variability in responses to this question indicates that some users understood this dynamic, while others did not. This could be improved with better training that includes best practices for communicating spatial intent depending on the situation.

## 6 Discussion

The results from this user evaluation validate the flexible and intuitive design of the RINAO user interface for information-based tasking, as well as the computational efficiency of the inference algorithm. Evaluation with the 10 public safety experts reflects a positive perspective on the developed interface and its capability to transform the nature of a piloting task. Subjects reported that they could focus more on the searching aspect of their assignment rather than on piloting the aircraft, reflecting effective task allocation within the human–robot team. While future work is needed to evaluate this interface’s effectiveness in conjunction with an underlying execution algorithm, providing operators with a reliable modality that allows them to focus on higher-level cognitive functions represents a significant improvement over current methods.

Each of the 10 operators engaged with the interface in a unique approach, with a selection of their inputs shown in Figure 5. As previously discussed, some operators provided very sparse inputs, such as Subject 1 (S1), whereas others added significant detail across the operational area, such as Subject 2 (S2). Ensuring that this system is flexible to a diversity of user approaches is especially important when implementing systems for context-specific information tasking. However, two subjects noted that they found some aspects of the inputs confusing as there were multiple ways to convey the same direction. For example, when specifying the assigned task of flying in the southern valley, different users sketched the northern valley area as a no-fly-zone, shown by S2, and gave observations to “Go Inside” areas of the user-prioritized southern valley, broadly shown by S8, S1, and S3, or to “Don’t Go Inside” the prioritized northern valley as shown by S10. As each of these three inputs is treated equally within the algorithm, users need to develop an understanding of the algorithmic structure, which can be improved through additional training with the system and the definition of general best practices.

Enabling operators to clearly visualize the system’s interpretation of their inputs can be especially helpful for system users to build an understanding of the results of the underlying algorithm. In agreement with previous work by Wang and Lau (2023) and Tabrez et al. (2020), displaying the visualized reward map proved especially useful to users, with one subject stating “it is super helpful to see the [reward] visualization.” While these types of visual feedback displays are common, this contribution focuses on RINAO’s ability to rapidly update the reward based on novel inputs and additional features in the environment. Users often provided multiple rounds of inputs and observed how the system responded before adding additional information. Subject 9 iterated on their inputs 17 times before being satisfied with their approach, yet they still commented that “they might redo it,” having learned how the system interpreted their information. Enabling this type of bi-directional feedback is critical for achieving human–robot mental model alignment, and previous work by Burks et al. (2023) has shown that it improves overall trust. In addition, showing this visual display allowed users to notice features that they were previously unaware of, such as highlighted geographic flow lines that were not explicitly included on the given map. Depending on the downstream planning and execution, visualizing the resulting system’s mental model is critical to implementing a predictable and, therefore, trustworthy system.

In addition to enabling effective interactions, we also show that RINAO presents a data- and time-efficient method of inferring geospatial value compared to an IRL baseline. Despite using an average of 1.5% of the data of the IRL approach, RINAO resulted in an equal or better value alignment across our five different evaluation metrics. Within the four Sanneman metrics, RINAO matches the IRL baseline in *Feature Alignment* and slightly outperforms the baseline in Policy Alignment. However, RINAO significantly outperforms IRL in the location rating, which may point to some deficiencies in the Sanneman approach’s consideration of reward alignment for methods of inference that account for uncertainty in the resulting estimate.

RINAO’s substantially smaller data requirements improve the system’s usability for the operator. In this particular application, asking users to give a specific trajectory that they would follow proved challenging. One subject remarked, “It’s hard to choose a single flight path that I would follow.” Operators engaged in the searching task often dynamically adjust their trajectories due to the complexity of the underlying terrain and its impact on aircraft performance. While inference over the resulting trajectories that the aircraft might follow allows for effective inference in the resulting geospatial value, RINAO directly asks operators what they are interested in, leading to more intuitive and directed inputs. In addition, the RINAO interface could be naturally extended to include trajectory inputs from users without requiring modifications to the algorithm. RINAO still maintains a number of hyperparameters that must be hand-tuned to balance user inputs, but the IRL baseline contains significantly more hyperparameters and behavioral assumptions. If the mission needs change the requirements of the planning and execution module, such as switching to a unique search pattern or perimeter-patrolling behavior, RINAO provides a generalized interface that can adjust the underlying execution for geospatial information-based tasking.

### 6.1 Future work

In evaluating the feedback from our subjects and the resulting performance of our algorithm, several areas of improvement were identified. First, effectively briefing and training subjects on how to use the system led to successful outcomes. When users are taught how to use aircraft in public safety settings, they spend dozens of hours familiarizing themselves with the aircraft controls, operation, user interface, and associated complexity of integrating aerial operations into incidents. Given that most users spent less than an hour familiarizing themselves with the system, additional standardized training will likely improve human–robot value alignment. It was also discovered that more geographic features could be included within the database, particularly ridge lines. While this may depend on the specific scenario, incorporating more direct topographical elevation data can be valuable, and it is clear that RINAO is flexible in inferring distributions over many environmental features in the environment. Finally, the system’s usefulness in operations will depend on enabling various methods for downstream planning and execution based on the resulting inputs. A more autonomous approach, such as that described by Ray et al. (2024), should be complemented by additional, user-selected options that give varying levels of autonomy while maintaining high levels of human control. For example, this could include following a series of waypoints with geospatial value-based path optimization, such as following the curve in a road between two waypoints.

### 6.2 Limitations

This evaluation still presents certain limitations in its contributions. Working with real users presents challenges due to the diversity of perspectives, use cases, and assumptions that they bring to a system. As such, this study limits the generalization of these results to this specific user group of public safety experts. The performance of these users is also dependent on this specific scenario and the limited training that was provided. Further evaluation is needed to validate this approach with more extensive training and in a live, high-stakes scenario with a real aircraft with integrated autonomy. Specifically, regarding the approach, RINAO is dependent on the selection of a subsequent downstream planning method that can optimize over the user’s reward map. While the approach presented in Ray et al. (2024) offers a potential POMDP-based solution, several various optimization-based planners can also leverage this reward map. RINAO also limits the inputs to the provided set of points, priorities, and semantic observations. These inputs are arguably comprehensive in terms of describing desirable geospatial coverage, but future work can explore the addition of temporal commands to further refine the sequence of execution.

## 7 Conclusion

This work presents an effective interface for collaborative human–robot teaming in information-based tasking, with search and rescue as an operational modality. The RINAO approach combines an intuitive user interface with a structured algorithmic inference framework that invites users to provide direct input regarding desired autonomous behavior. Building upon geographic database information, which includes features such as trails and tree cover, users can opportunistically define new spatial features in the environment using a sketch-based interface. This allows users to naturally constrain the system’s autonomy to move near, north, or inside certain regions or landmarks in a way that reflects the communication patterns present in human teams. The interface is tested by 10 public safety experts with a combined 193 years of operational experience, which shows a high degree of usability and effective geospatial reward alignment. Compared to an inverse reinforcement learning baseline, RINAO accomplishes similar or better reward alignment and an order of magnitude improvement in algorithmic data and computational efficiency.

While public safety presented an attractive arena for testing our interface, it can be directly adapted into any type of mission that requires collaborative autonomy to address complex and nuanced information-based tasking that is dependent on geospatial features and values. Future work will implement the realized interface in conjunction with a particular aircraft and evaluate the combined human-robot team in a relevant training scenario.

## Data Availability

The raw data supporting the conclusions of this article will be made available by the authors, without undue reservation.

## Footnotes

1
https://www.thunderforest.com/maps/outdoors/

2
https://github.com/JuliaCI/BenchmarkTools.jl
